# Cardiomyocyte protein O-GlcNAcylation is regulated by GFAT1 not GFAT2

**DOI:** 10.1016/j.bbrc.2021.10.056

**Published:** 2021-12-17

**Authors:** Adam A Nabeebaccus, Sharwari Verma, Anna Zoccarato, Giulia Emanuelli, Celio XC. Santos, Katrin Streckfuss-Bömeke, Ajay M. Shah

**Affiliations:** a BHF Centre of Excellence King's College London, The James Black Centre, 125 Coldharbour Lane, London, SE5 9NU, UK; b German Centre for Cardiovascular Research, 10785 Berlin, partnersite Göttingen, Germany; c Institute of Pharmacology and Toxicology, University of Würzburg, 97078 Würzburg, Germany

**Keywords:** Hexosamine biosynthesis pathway, GFPT1, GFPT2, GFAT1, GFAT2, O-GlcNAc

## Abstract

In response to cardiac injury, increased activity of the hexosamine biosynthesis pathway (HBP) is linked with cytoprotective as well as adverse effects depending on the type and duration of injury. Glutamine-fructose amidotransferase (GFAT; gene name *gfpt*) is the rate-limiting enzyme that controls flux through HBP. Two protein isoforms exist in the heart called GFAT1 and GFAT2. There are conflicting data on the relative importance of GFAT1 and GFAT2 during stress-induced HBP responses in the heart.

Using neonatal rat cardiac cell preparations, targeted knockdown of GFPT1 and GFPT2 were performed and HBP activity measured. Immunostaining with specific GFAT1 and GFAT2 antibodies was undertaken in neonatal rat cardiac preparations and murine cardiac tissues to characterise cell-specific expression. Publicly available human heart single cell sequencing data was interrogated to determine cell-type expression. Western blots for GFAT isoform protein expression were performed in human cardiomyocytes derived from induced pluripotent stem cells (iPSCs).

GFPT1 but not GFPT2 knockdown resulted in a loss of stress-induced protein O-GlcNAcylation in neonatal cardiac cell preparations indicating reduced HBP activity. In rodent cells and tissue, immunostaining for GFAT1 identified expression in both cardiac myocytes and fibroblasts whereas immunostaining for GFAT2 was only identified in fibroblasts. Further corroboration of findings in human heart cells identified an enrichment of GFPT2 gene expression in cardiac fibroblasts but not ventricular myocytes whereas GFPT1 was expressed in both myocytes and fibroblasts. In human iPSC-derived cardiomyocytes, only GFAT1 protein was expressed with an absence of GFAT2.

In conclusion, these results indicate that GFAT1 is the primary cardiomyocyte isoform and GFAT2 is only present in cardiac fibroblasts. Cell-specific isoform expression may have differing effects on cell function and should be considered when studying HBP and GFAT functions in the heart.

## Highlights

• GFAT2 is not expressed in cardiac myocytes.
• GFAT2 is abundantly expressed in cardiac fibroblasts.
• GFAT1 is expressed in both cardiac myocytes and fibroblasts.
• GFAT1 regulates the stress-induced increase in cardiac O-GlcNAcylation.

## 1 Introduction

The hexosamine biosynthesis pathway (HBP) converts fructose 6-phosphate from glycolysis into the end-product UDP-N-acetylglucosamine (UDP-GlcNAc) which is required for a range of important macromolecule glycosylation such as the O-GlcNAcylation of many proteins. The key enzyme responsible for making fructose 6-phosphate into glucosamine 6-phosphate and thus committing it to the HBP is glutamine-fructose amidotransferase (GFAT; gene name *gfpt*). It is the first and rate-limiting step of this pathway, transferring the amide group from glutamine to fructose and forming glucosamine 6-phosphate. This is subsequently converted to UDP-GlcNAc by incorporating acetyl-CoA and UTP through successive steps.

There are two protein isoforms of GFAT called GFAT1 and GFAT2, encoded by separate genes, *gfpt1* and *gfpt2* respectively, and located on different chromosomes [1,2]. They share 75% homology and differ in important ways, namely regulation by phosphorylation [[3], [4], [5]], gene expression [[6], [7], [8]] and tissue distribution [9]. Genetic mutations affecting GFAT1 and leading to loss of function are primarily characterised by a muscular disorder called congenital myasthenic syndrome [[10], [11], [12]]. Another interesting difference between the isoforms is that GFAT1 is present as a splice variant in striated muscles termed long and short variants [[13], [14], [15]]. The significance of GFAT1 splice variants in striated muscle remains unclear. In contrast, GFAT2 deficiency has been recently described to be associated with a completely unrelated phenomenon, asthenozoospermia [16] with no other specific disease manifestation reported. *Gfpt*-specific SNPs have been associated with risk of diabetes and their complications but the results are inconsistent [[17], [18], [19], [20]]. Increased activity of both isoforms has been noted in driving carcinogenesis in a variety of tissues [[21], [22], [23], [24], [25]].

Despite these differences noted in isoform tissue distribution, regulation, and disease phenotype relatively little is known about their role in the heart. Cardiac HBP activity is observed to be elevated in a variety of stress situations including pressure-overload, myocardial infarction, and diabetic cardiomyopathy; however, the pathological role of HBP is unclear [[26], [27], [28], [29], [30]]. The consensus from early studies suggested that GFAT2 was the predominant isoform expressed in mammalian hearts [[31], [32], [33], [34]]. However, these studies were not consistent in the reporting of GFAT1 either at transcriptional or protein levels. More recent studies have indicated a specific role for GFAT1 rather than GFAT2, suggesting differences in their regulation and functions [[35], [36], [37]]. It remains unclear why these studies differ in their reports regarding GFAT isoform functions in the heart.

We now report that GFAT protein expression in the heart is cell-specific. GFAT2 is in fact absent in rodent and human cardiomyocytes and only expressed in fibroblasts, whereas GFAT1 is present in both cell types. Cell-specific differences in isoform expression likely account for the prior conflicting data and indicate a need to evaluate cell-specific functions.

## 2 Materials and methods

### 2.1 Materials

Primary antibodies for immunoblotting and immunofluorescence were: GFAT1 (Cell signalling - rabbit monoclonal D12F4; Abcam - ab125069, rabbit monoclonal), GFAT2 (Abcam - ab190966, rabbit monoclonal), O-GlcNAc (mouse monoclonal RL2; Abcam), Troponin T (Abcam - ab8295), PDGFR alpha (R&D Systems - AF1062), alpha-actinin (Sigma), GAPDH (Sigma). Secondary antibodies were: LI-COR IRDye donkey anti-rabbit IR800, donkey anti-mouse IR680, donkey anti-mouse IR800, donkey anti-rabbit IR680, donkey anti-goat IR 680. For Immunofluorescence: AlexaFluor 488 goat anti-rabbit, 546 goat anti-mouse, 633 donkey anti-goat.

### 2.2 Cell culture and transfection

Neonatal rat cardiac cells were isolated from 1 to 2 days old Sprague-Dawley rats. Isolated cells were plated in DMEM media containing M199 (Sigma), 10% FBS,1% Streptomycin/Penicillin. After 16–24 h, cells were washed twice with serum-free media and kept at 37 °C. Cells were then transfected overnight with *Gfpt1* FlexiTube siRNA or scramble siRNA (Qiagen). Cells were treated with phenylephrine (100 μM) for 24 h before harvesting.

### 2.3 Western blots

Total protein was extracted from cells and tissue with a lysis buffer (25 mM Tris/HCl, 150 mM NaCl, 2 mM EGTA, 5 mM EDTA, 0.5% Nonidet P-40, final pH 7.2) containing protease inhibitors and PUGNAc (80 μM). Protein concentrations were determined using BCA Protein Assay kit (ThermoFisher). 20 μg of protein was loaded onto 1.0 mm 4–12% Bis-Tris plus gels (Invitrogen) and transferred onto nitrocellulose membranes (Amersham 0.45 μM, GEHealthcare). Membranes were blocked in 4% milk and incubated with primary antibody. Bound primary antibodies were further incubated with fluorescent dye labelled secondary antibodies detected by an Odyssey infrared image scanner (Li-Cor). All primary antibodies were used at 1:1000 and secondary antibodies at 1:15000 dilutions.

### 2.4 Histology and immunofluorescence

Cells were washed twice in PBS and fixed in 7.5% formalin for 10 min at room temperature. Cells were washed and permeabilized with 0.05% Triton at room temperature for 3 min. After three washes with PBS, cells were incubated with primary antibody in a buffer containing 3% BSA and 1:50 normal goat serum. Cells were washed three times in PBS before incubating with Alexafluor antibody for 1 h at room temperature. Cells were washed again thrice with PBS and incubated with DAPI (1 mg/ml) for nuclear staining. Stained cells were mounted using Mowiol at room temperature overnight and imaged using a Nikon Eclipse Ti Inverted Spinning Disk Confocal System. All images were obtained using a 60X objective and were analysed using Image J 1.5 software.

Heart tissues were fixed in 4% paraformaldehyde for 24 h and dehydrated in 70% ethanol. Tissue sections were de-paraffinized and rehydrated with successive changes of xylene, ethanol and water. Tissue sections were permeabilized and incubated with 0.4% Triton in PBS and incubated in blocking buffer containing 3% normal goat serum.

Studies were conducted in accordance with the UK Home Office Guidance on the Operation of Animals (Scientific Procedures) Act, 1986 and with institutional ethics committee approval.

### 2.5 Data mining of public database

The publicly available database on large-scale single-cell and single-nucleus transcriptomes from adult human hearts [38] was interrogated using the online platform available at www.heartcellatlas.org/. Using the interactive viewer for cardiomyocyte and fibroblast populations, visualisations for *Gfpt1* and *Gfpt2* fold-change expression values were generated in the form of t-SNE plots.

### 2.6 Statistical analysis

All data are shown as the mean ± SEM. 2-way ANOVA was used to compare differences in means, followed by a post-hoc test for multiple comparisons. *P* < 0.05 was considered significant. Analyses were undertaken using GraphPad Prism (v.8.4.3).

## 3 Results

### 3.1 GFAT1 not GFAT2 regulates HBP activity in cardiac cells

**Fig. 1:**
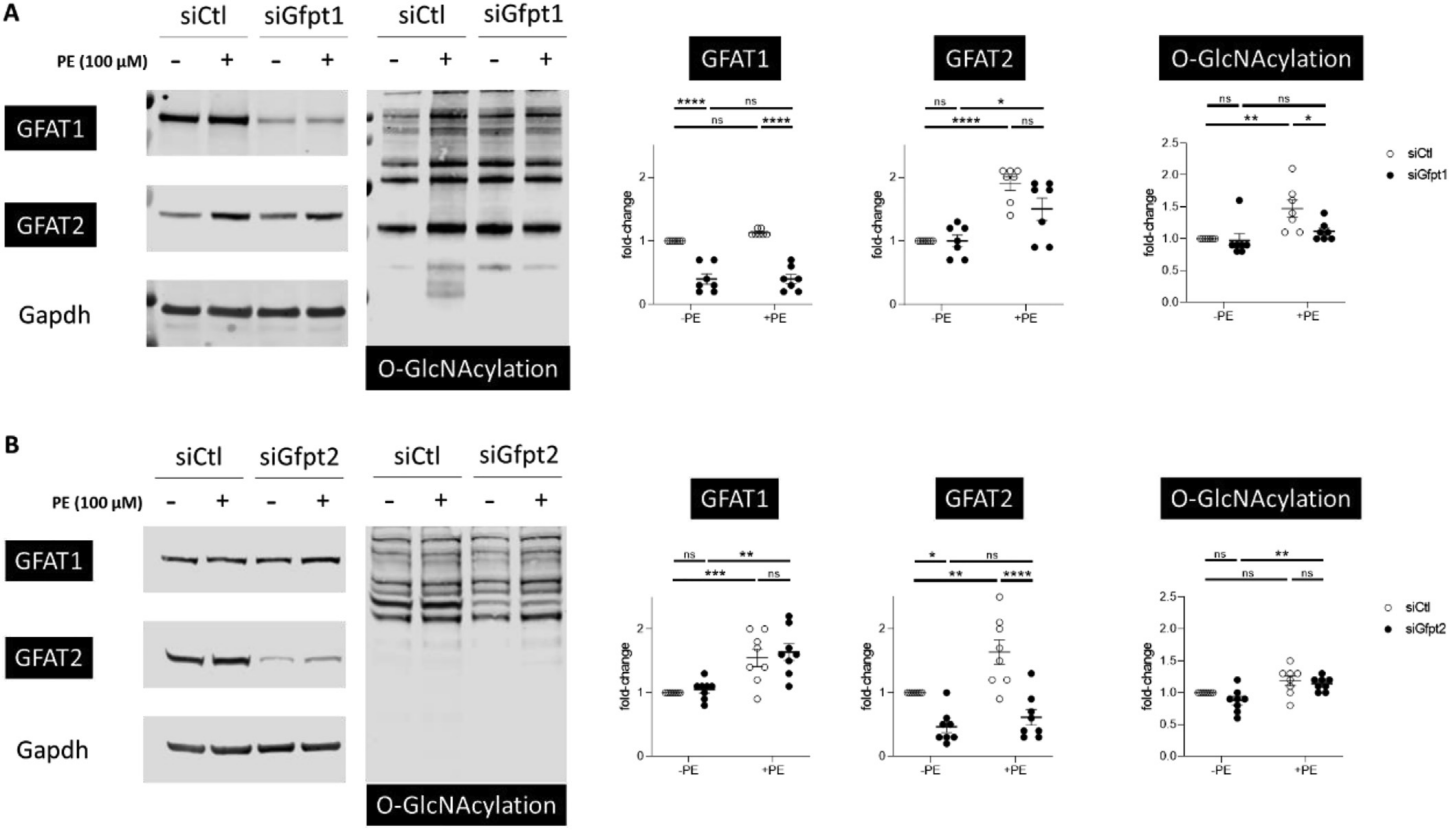
**A.** Western blot of neonatal rat cardiac cell preparations with knockdown of *Gfpt1* with siRNA with and without treatment with 100 μM phenylephrine (PE). *Gfpt1* knockdown was specific for GFAT1 protein without affecting GFAT2 expression levels. GFAT1 knockdown blunted the PE-induced increase in O-GlcNAcylation. **B.***Gfpt2* silencing using siRNA was specific for GFAT2 isoform but its knockdown did not prevent PE-induced O-GlcNAcylation. (7-8 separate experiments per group, 2-way ANOVA; *p*-values corrected for multiple comparisons).

The GFAT-catalysed reaction is the rate-limiting step for UDP-GlcNAc synthesis. UDP-GlcNAc can be attached to serine or threonine residues on a variety of intracellular proteins. This modification is known as O-GlcNAcylation and is a marker for increased HBP activity. This modification is readily detected by antibodies that recognise O-GlcNAc-modified proteins. HBP activity and O-GlcNAcylation is observed to be increased in hearts subject to hypertrophic stimuli. To further ascertain GFAT isoform expression in cardiac cells, we performed Western blots of rat primary cardiac cell preparations treated with phenylephrine (PE) stress to recapitulate hypertrophic stress. These preparations typically contain cardiac myocytes and a small proportion of cardiac fibroblasts and thus constitute a mixture of “cardiac cells”. Using western blotting with specific monoclonal antibodies against GFAT1 and GFAT2, we demonstrate that we could specifically knockdown *Gfpt1* and *Gfpt2* using siRNA (Fig. 1 A and B). In addition, we observed both expression of GFAT1 and GFAT2 at basal conditions with both isoforms showing an increase in expression with PE-stimulation. Interestingly, only *Gfpt1* knockdown significantly attenuated the PE-induced increases in protein O-GlcNAcylation between the two isoforms (Fig. 1A). *Gfpt2* knockdown had no effect on PE-induced increases in O-GlcNAcylation (Fig. 1B). This confirmed that GFAT1 was the primary isoform that regulates HBP activity in cardiac cells.

### 3.2 GFAT isoform expression differs between cardiomyocytes and fibroblasts

**Fig. 2:**
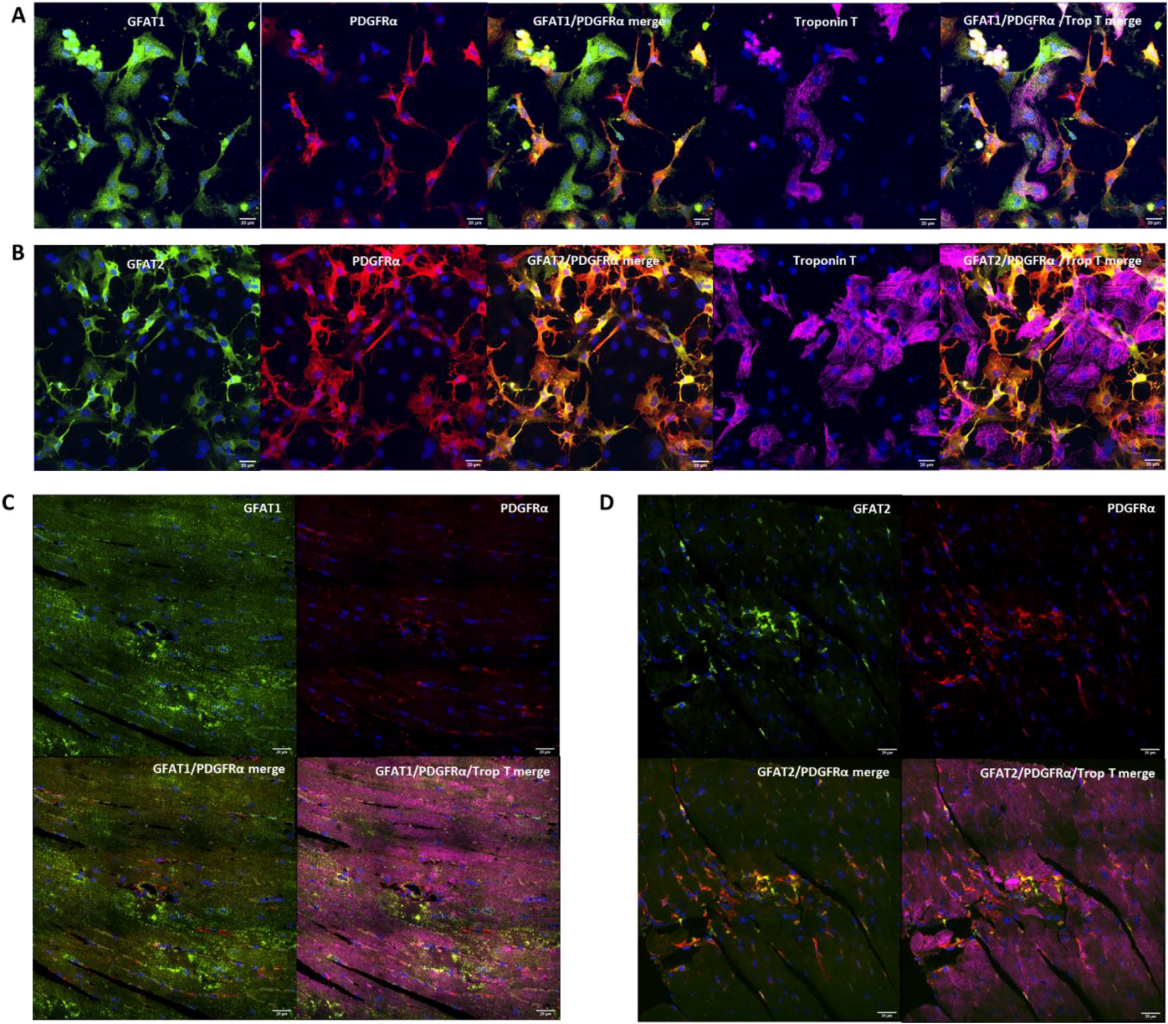
**A.** Immunofluorescence staining of neonatal rat cardiac preparations with a GFAT1-specific antibody (green) which is shown expressed in cardiomyocytes (far-red; troponin T) and fibroblasts (red; PDGFRα). Nuclei stained with DAPI (blue). Scale bar, 20 μm. **B.** Immunofluorescence staining of neonatal rat cardiac preparations with a GFAT2-specific antibody (green). GFAT2 is only expressed in fibroblasts (red; PDGFRα) and absent from cardiomyocytes (far-red; troponin T). Scale bar, 20 μm. **C.** In murine heart tissue sections, GFAT1 (green) stains both cardiomyocytes (far-red; troponin T) and fibroblast cells (red; PDGFRα). Nuclei stained with DAPI (blue). Scale bar, 20 μm. **D.** In murine heart tissue sections, GFAT2 only stains fibroblast cells (red; PDGFRα) and is absent from cardiomyocytes (far-red; troponin T).

To further understand the regulatory differences between GFAT1 and GFAT2, we used immunofluorescent microscopy to determine the cellular expression of each isoform. In cultured primary cardiac cells, we could identify both cardiomyocyte and fibroblast staining for GFAT1 (Fig. 2 A). However, GFAT2 staining was limited to fibroblasts and absent in cardiomyocytes (Fig. 2 B). We performed immunofluorescent microscopy of murine adult hearts and observed that GFAT1 was expressed abundantly in both cardiomyocytes and fibroblasts (Fig. 2C) whereas GFAT2 was only present in fibroblasts (Fig. 2 D). These findings support the previous data indicating that GFAT1 is the primary cardiomyocyte isoform and is responsible for stress-induced increases in O-GlcNAcylation.

### 3.3 Human cardiomyocytes express GFAT1 but do not express GFAT2

**Fig. 3:**
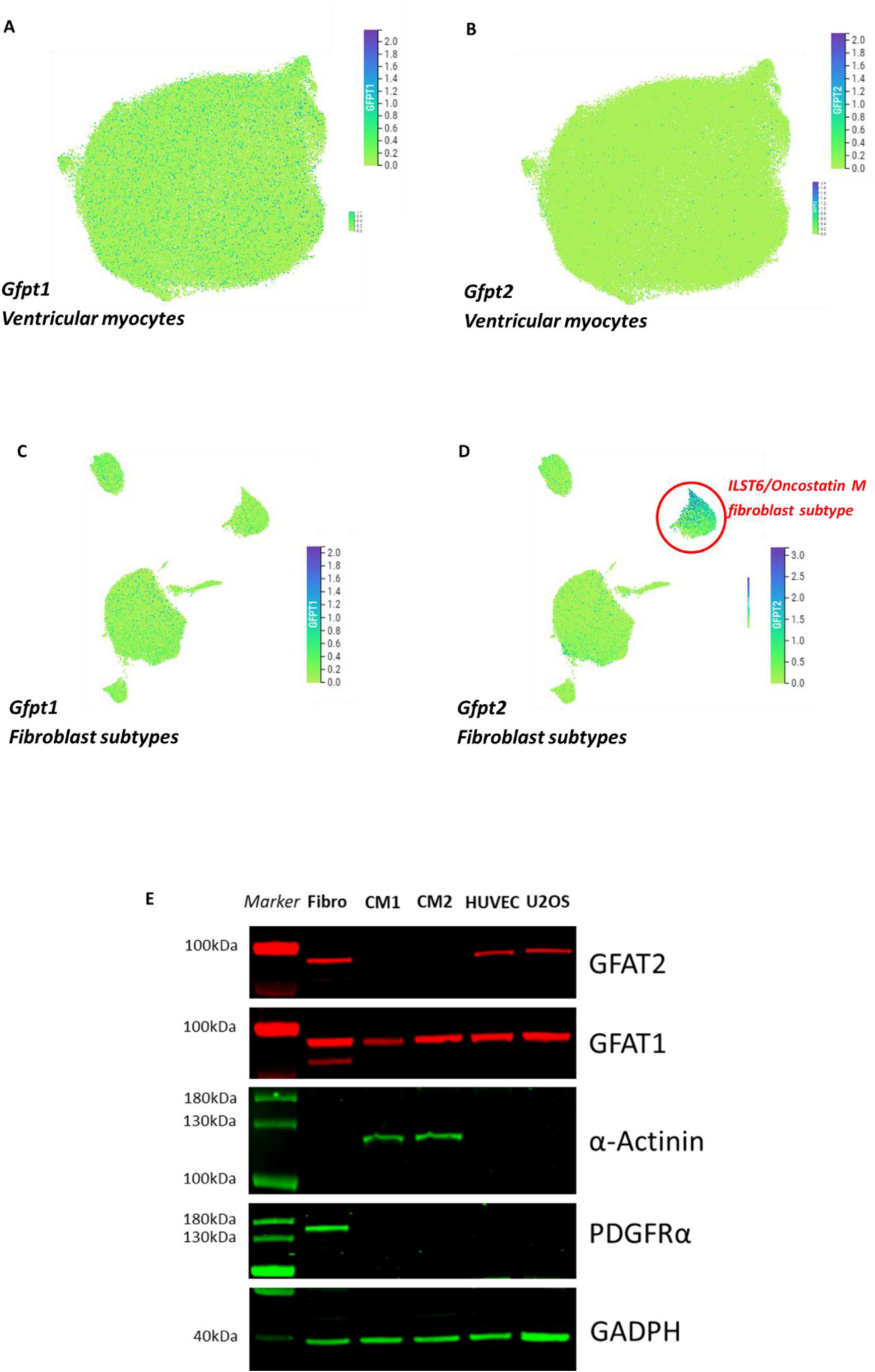
**A**. t-distributed stochastic neighbour embedding (*t*-SNE) plot showing relative fold expression levels of *Gfpt1* in human ventricular myocytes. Green to blue indicate 0 to 3-fold increase expression differences. B. *t*-SNE plot for Gfpt2 in human ventricular myocytes. **C.** t-SNE plot for cardiac fibroblast subpopulations showing Gfpt1 expression levels. **D.** t-SNE plot for cardiac fibroblast subpopulations showing enrichment in a subpopulation characterised by ILST6/Oncostatin M signalling (encircled in red). **E.** Western blot of adult-human iPSC-derived cardiomyocytes (CM1 and CM2 are separate differentiations and late-stage maturation) showing expression of GFAT1 but not GFAT2. Comparison with other cell types to show GFAT isoform specificity (Lane 1 – Fibro; human foreskin fibroblast cell line, Lane 2 – CM1, Lane 3 – CM2, Lane 4 – HUVEC; human umbilical vein endothelial cell, Lane 5 – U2OS; human osteosarcoma cell line. α-actinin is specific for cardiomyocytes, PDGFRα is specific for fibroblasts).

To determine if our findings of cell-type specific expression of GFAT isoforms in rodent cardiac cells and tissue also apply to human hearts, we turned to publicly available single cell RNA sequencing data made available in the Human Heart Atlas [38] (www.heartcellatlas.org/). t-SNE plots demonstrate that *Gfpt1* is expressed in ventricular myocytes (Fig. 3 A), whereas *Gfpt2* is absent to minimally expressed in cardiomyocytes (Fig. 3 B). Turning to fibroblasts, the human cell atlas characterised subpopulations of fibroblasts determined by their gene expression profiles. *Gfpt1* was expressed in most fibroblast subpopulations (Fig. 3C). Interestingly, *Gfpt2* was highly enriched in a subpopulation of human fibroblasts characterised by their higher expression of ILST6/Oncostatin-M receptor signalling pathway genes but lower expression of ECM-related genes (Fig. 3 D). This expression data fits our findings of protein level data in rodent cardiac cells and tissue. To further establish protein level expression, we took human cardiomyocytes derived from iPSCs and compared them to other human cell types known to express both forms of GFAT. We found only GFAT1 to be expressed in cardiomyocytes and that GFAT2 was absent (Fig. 3 E). This supports the sequencing data from human hearts as well as demonstrates that the cell-specific expression pattern is conserved across mammalian species.

## 4 Discussion

Both GFAT1 and GFAT2 are known to be expressed in cardiac tissue; however very little is known about their relative expression in cell types that make up cardiac tissue. It has been assumed that both isoforms are expressed in cardiomyocytes with previous studies indicating that GFAT2 was the major isoform expressed in the heart. However, no studies have previously addressed this at the cellular level, and we believe this is the first study to demonstrate an isoform difference in cell-type expression at the protein level.

Several notable findings are reported in this study. Firstly, GFAT2 is not expressed in cardiomyocytes. Secondly, GFAT2 is expressed in fibroblasts and may be enriched in sub-populations of fibroblasts. Thirdly, GFAT1 is present in both cardiomyocytes and fibroblasts and finally that GFAT1 is responsible for stress-induced increases in cardiac total protein-O-GlcNAcylation. We consider these findings may go someway in explaining differences in HBP regulation and activity in the heart and provide nuanced information on the role of GFAT-HBP signalling and the cardiac response to disease. Several studies have identified specific regulatory features of GFAT1 both at gene expression level and through secondary modifications primarily by phosphorylation. An interesting feature of GFAT1 is its stress-responsive expression observed in the heart under conditions of pressure-overload and ischaemia-reperfusion [26,36,37]. Increasing GFAT1 activity has been linked to cell-survival pathways by increasing HBP activity and promoting cellular protein quality control [36,39]. Signifying its importance in striated myocytes are the reported myopathy in patients with *Gfpt1* deficiencies, some of whom may have an associated cardiac feature [40]. This lends support to the data that GFAT1 may be the dominant isoform in cardiac myocytes as identified in our study where it is subject to several forms of regulatory control.

In contrast we have not found GFAT2 to be expressed in myocytes but rather abundantly expressed in non-myocytes such as fibroblasts. Of significance, studies have found that GFAT2 is not activated by the stress-response in hearts which appears to be a specifically GFAT1-related phenomenon [35,41]. Given its abundant and specific enrichment in fibroblasts, it is curious to note that cardiac fibroblast-specific GFAT2 expression has not been characterised before and requires further work to establish its impact on cardiac physiology and disease. However, our finding that GFAT2 is localised to the cardiac fibroblast population may explain why earlier studies identified GFAT2 abundantly in heart tissue.

It is important to state that it is possible that GFAT2 expression could be induced in cardiomyocytes during hypertrophic stimulation or indeed augmented further in fibroblasts leading to increased O-GlcNAcylation. Our data do not differentiate between this possibility and further work in a hypertrophic model or PE-treated fibroblast preparations would be needed to examine these possibilities.

In conclusion, identifying a difference in protein isoform expression depending on cardiac cell type has important implications as their regulation and cellular effects appear to be different. GFAT1 expression is stress-responsive and may promote cell survival in some circumstances. GFAT2 protein expression appears to be absent from cardiac myocytes and as previously published does not appear to be regulated by cardiac stress-responses. However, it is seemingly abundant at both transcript and protein level in cardiac fibroblasts where its role requires further investigation. Noting the marked differences in cell-type expression and regulatory properties of GFAT isoforms in the heart, it is likely the subsequent activation of HBP may have functional differences at the cell level that may impact on overall cardiac tissue properties depending on the type of pathology. Further work should focus on GFAT isoform expression differences between cardiac myocytes and fibroblasts and how this impacts on cardiac biology.

## Declaration of interests

The authors declare that they have no known competing financial interests or personal relationships that could have appeared to influence the work reported in this paper.
